# Evaluation of Peripapillary Microcirculation in Patients with Acromegaly

**DOI:** 10.14744/bej.2021.48343

**Published:** 2021-12-17

**Authors:** Mine Karahan, Atilim Armagan Demirtas, Leyla Hazar, Sedat Ava, Zafer Pekkolay, Ugur Keklikci

**Affiliations:** 1. Department of Ophthalmology, Dicle University Faculty of Medicine, Diyarbakir, Turkey; 2. Department of Ophthalmology, Health Sciences University, Izmir Tepecik Training and Research Hospital, Izmir, Turkey; 3. Department of Endocrinology and Metabolism, Dicle University Faculty of Medicine, Diyarbakir, Turkey

**Keywords:** Acromegaly, optical coherence tomography angiography, optic nerve head, vascular density

## Abstract

**Objectives:**

The aim of this study was to evaluate the radial peripapillary capillary (RPC) and the optic nerve head (ONH) perfusion of patients with acromegaly using optical coherence tomography angiography (OCTA).

**Methods:**

Twenty-four eyes of 24 acromegaly patients comprised the study group and 24 eyes of 24 healthy individuals were used as a control group. The ONH and RPC vascular density (VD) was measured for each patient using OCTA. The insulin-like growth factor 1 (IGF-1) levels were also recorded and compared.

**Results:**

The VD of the inferior nasal ONH and nasal RPC was significantly lower in the acromegaly group than in the control group (p=0.047 and p=0.001, respectively). There was a significant negative correlation between the VD of the superior nasal ONH and the IGF-1 level (r=-0.283, p=0.038).

**Conclusion:**

The ONH and RPC VD values measured using OCTA were segmentally different in the acromegaly group compared with those of the control group. This method of non-invasive quantitative analysis of retinal perfusion using OCTA may be useful for future studies involving patients with acromegaly.

## Introduction

Acromegaly is a disease characterized by an increase in insulin-like growth factor 1 (IGF-1) and growth hormone (GH), and it is usually caused by a pituitary adenoma. Common comorbidities associated with acromegaly include hypertension, diabetes, bone and joint disorders, skin thickening, and bitemporal hemianopsia (1, 2). IGF-1 and its receptors are distributed differently in the retina than in other tissues (3).

Harvey et al. (4) reported that GH is found in the human retina and vitreous fluid and may play a role in certain ocular disorders. It has also been reported that GH receptor insensitivity, known as Laron syndrome, causes a decrease in retinal vascularity and that GH deficiency is associated with optic nerve hypoplasia (5).

By optical coherence tomography angiography (OCTA), it is possible to quantitatively evaluate retinal and choroidal microvascular circulation in a non-invasive manner without using dyes (6). In this study, we aimed to compare the peripapillary microcirculation of patients with acromegaly with that of healthy controls by OCTA.

## Methods

In this observational case–control study, 24 right eyes from 24 patients with acromegaly who underwent operation for pituitary adenoma and were observed in the endocrinology and metabolism clinic were evaluated in the ophthalmology clinic. Furthermore, 24 right eyes from 24 healthy individuals, who were similar in terms of age and gender, were also included in the study as controls. The study was conducted in accordance with the Declaration of Helsinki and approved by the Ethics Committee of (date: January 7, 2021, number: 61), and written informed consent was obtained from all patients included in the study.

A complete ophthalmological examination, including best-corrected visual acuity, biomicroscopic evaluation, intraocular pressure measurement using a non-contact tonometer, and a fundus examination, was performed for all eyes included in the study. Patients with systemic diseases (e.g., diabetes mellitus and hypertension), ocular diseases (e.g., glaucoma and maculopathy), and other diseases (such as those that would impair the optic nerve evaluation and optic disc anomalies) were excluded from the study, along with those who had previously undergone intraocular surgery.

All patients had previously undergone operation for pituitary macroadenoma, and they underwent follow-up in the endocrinology and metabolism clinic. We did not include patients who used any drugs in the study. IGF-1 levels (ng/mL) and all other metabolic parameters were measured using immunoradiometric tests. In addition, age-adjusted reference ranges were used to evaluate IGF-1 levels.

### OCTA Measurement

OCTA (RTVue XR Avanti, Optovue Inc., Fremont, CA, USA) images were generated by an experienced technician. The optic nerve head (ONH) and radial peripapillary capillary (RPC) vascular density (VD) values (%) were recorded. Images with signal strengths >50 without any segmentation errors or motion artifacts were included in the study.

### Statistical Analysis

Statistical analysis was performed using Windows version 21.0 and Statistical Package for the Social Sciences (SPSS) software (SPSS Inc., Chicago, IL, USA). All data are expressed as means ± standard deviations. For variables in a group, compliance with normal distribution was determined using the Shapiro–Wilk test, while an independent t-test or Chi-square test was used to compare variables between groups. Finally, Pearson’s correlation test was used to evaluate the relationships. P-values of <0.05 were considered statistically significant.

## Results

**Figure 1.**
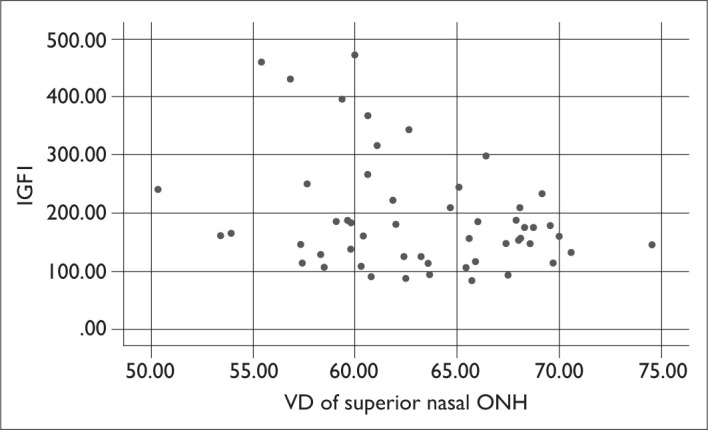
Scatter plot graph of correlation analysis of IGF-1 and ONH VD. Scatter plot graph of the correlation analysis of insülin-like growth factor 1 (IGF-1) level and superior nasal optic nerve head (ONH) vascular density (VD) (Pearson’s correlation; r=-0.283, p=0.038).

There was no significant difference between the study and control groups in terms of age or gender (p=0.073 and p=0.493, respectively). IGF-1 levels were significantly higher in patients with acromegaly (p<0.001). The mean follow-up period was 8.08±4.67 years in acromegaly group. Demographic characteristics and serum IGF-1 levels of the study participants are presented in Table 1. The signal strength of the OCTA images was similar between the two groups (acromegaly group: 75.96±9.84; control group: 77.58±9.85; p=0.549). Furthermore, inferior nasal ONH VD and nasal RPC VD were significantly lower in the acromegaly group (p=0.047 and p=0.001, respectively). However, no significant differences were observed between patients with acromegaly and control patients in terms of ONH VD and RPC VD values in other segments (Tables 2 and 3). Moreover, a significant negative correlation was found between IGF-1 level and superior nasal ONH VD (r=–0.283, p=0.038, [Fig. 1]).

**Table 1.** Demographics and serum IGF-1 levels of acromegaly and control groups

	**Acromegaly (n=24)**	**Control (n=24)**	p-value^*^
	**Mean±SD**	**Mean±SD**	
Age, years	43.12±11.42	37.75±8.70	†0.073
Gender, female/male	9/15	11/13	^*^0.493
IGF-1, ng/mL	246.77±117.23	140.20±23.81	^†^ **<0.001**

^†^Independent t-test, ^*^Chi-square test. Bolded values represent significant p<0.05. IGF-1: Insulin-like growth factor 1; SD: Standard deviation.

**Table 2.** Comparison of optic nerve head (ONH) vascular density values between the groups

**ONH vascular density (%)**	**Acromegaly (n=24)**	**Control (n=24)**	p-value^*^
	**Mean±SD**	**Mean±SD**	
Whole image	60.81±3.37	60.87±3.07	0.943
Inside disc	58.17±5.56	56.70±4.21	0.307
Peripapillary	65.11±2.82	64.12±2.94	0.241
Nasal	63.86±3.17	62.92±4.19	0.388
Inferior nasal	62.92±4.19	65.92±5.85	**0.047**
Inferior temporal	66.08±6.59	66.48±4.33	0.803
Superior temporal	66.07±4.95	65.16±4.69	0.517
Superior nasal	65.05±4.58	63.48±5.00	0.263
Temporal	65.09±3.50	63.48±4.09	0.154

^†^Independent t test, Bolded values represent significant p<0.05. SD: Standard deviation.

**Table 3.** Comparison of RPC vascular density values between the groups

**RPC vascular density (%)**	**Acromegaly (n=24)**	**Control (n=24)**	p-value^*^
	**Mean±SD**	**Mean±SD**	
Whole image	59.18±4.05	59.03±3.05	0.883
Inside disc	50.31±8.92	50.39±6.51	0.970
Peripapillary	65.92±2.93	65.75±2.89	0.848
Nasal	63.50±3.49	67.26±3.78	**0.001**
Inferior nasal	65.81±5.78	67.26±3.78	0.308
Inferior temporal	67.77±7.75	68.58±4.05	0.654
Superior temporal	69.11±4.01	68.42±4.75	0.590
Superior nasal	65.08±4.93	63.38±5.18	0.251
Temporal	66.64±3.49	65.22±4.13	0.207

^†^Independent t test, Bolded values represent significant p<0.05. SD: Standard deviation; RPC: Radial peripapillary capillary.

## Discussion

The results of our study reveal that peripapillary microcirculation (detected by OCTA) of patients suffering from acromegaly affects the inferior nasal and nasal segments. The previous studies have suggested that acromegaly due to macroadenomas may cause segmental thinning in the retinal nerve fiber layer (RNFL) due to chiasmal compression (7-9). Macroadenomas, known to be the most common cause of chiasmal compression, can cause ischemic and mechanical damage to the crossed nasal retinal fibers (10).

Garcia et al. (11) suggested that the nasal quadrant RNFL thickness may be the optimal parameter for gauging peripheral visual field improvement after surgery to reduce compression in the optic chiasm. Cennamo et al. (12) reported that pituitary macroadenomas can cause thinning of the ganglion cell complex and peripapillary RNFL without chiasmal compression. The authors suggested that this may have been because a possible micro-compression was missed by magnetic resonance imaging (12).

While pituitary macroadenomas may cause visual symptoms depending on their size and disease duration, these symptoms are generally uncommon because macroadenomas are confined to the sella. Mechanical and ischemic damage to the chiasma fibers affect the nasal retinal fibers and cause visual field defects (11, 12). One small study showed decreased peripapillary vessel density in areas corresponding to visual field defects (13).

Numerous studies have reported the impairment of microvascular function in patients with active acromegaly (14-16). The most common change caused by acromegaly, depending on IGF-1 level, is microvascular wall hypertrophy. It is thought that there may be an association between IGF-1 level and microvascular dysfunction (14, 15). It has been suggested that GH and/or IGF-1 may directly or indirectly contribute to ocular dysfunction, which includes glaucoma and retinopathy (4). GH can cause an increase in the plasma level of von Willebrand factor, a marker of endothelial dysfunction (16). These effects may reflect the influence of GH on hyperglycemia and hyperlipidemia and may intensify during thrombus formation, the induction of von Willebrand factor, and endothelial dysfunction that causes retinopathy (4).

Furthermore, it has been found that IGF-1 levels in subretinal fluid are extremely high in patients with acromegaly (2, 3). Type 1 and Type 2 IGF receptors are present in cultured retinal pigment epithelial cells (17, 18).

In a study examining changes in the peripheral microcirculation of patients suffering from acromegaly, it was observed that the number and length of the capillaries were significantly lower (19). Chanson et al. (20), by directly measuring brachial artery hemodynamics, found lower regional blood flow and increased local resistance in patients with acromegaly. This may potentially be due to endothelial dysfunction and/or an arterial tonus disorder. In another study, when the eyes of patients with acromegaly were compared to the eyes of control participants in terms of OCTA parameters, significant decreases were found in the central vessel density and central perfusion density values in all regions in the acromegaly group (21). The aim of the present study was to evaluate ONH VD and RPC VD in patients with acromegaly undergoing surgery for macroadenoma. It was found that the ONH VDs in inferior nasal and RPC VDs in nasal segments in the acromegaly group were lower than in controls.

This study has certain limitations. First, this was a cross-sectional study; therefore, we cannot comment on the value of VD measurements for evaluating pre- and post-operative changes, relationship with adenoma size, or changes in VD values. A second limitation is the small sample size, which was due to the coronavirus disease 2019 pandemic. Third, segmental vessel density impairment cannot be explained by a high plasma level of IGF-1. If that was the case, one would expect the impairment to be generalized. This may be related to interference in axoplasmic flow induced by compression and local ischemia at the level of the optic chiasm. This can also be shown by visual field examination. If the study design was a pre- and post-operative vessel density and visual field examination, perhaps an increase in vessel density would be detected together with visual field improvement.

## Conclusion

İt was found that the ONH VDs in inferior nasal and RPC VDs in nasal segments, measured by OCTA in the acromegaly group, were lower than in healthy controls. Importantly, this may demonstrate the compression effect at a microvascular level, as opposed to a visual field defect, where the effect can be seen directly. In addition, non-invasive quantitative retinal perfusion analyzes by OCTA may be useful in future studies involving acromegaly patients.

### Disclosures

**Ethics Committee Approval:** Dicle University Ethics Com- mittee, protocol number: 61, Date: 07/01/2021.

**Peer-review:** Externally peer-reviewed.

**Conflict of Interest:** None declared.

**Authorship Contributions:** Involved in design and conduct of the study ( MK, AAD, LH, ZP), preparation and review of the study (MK, AAD, LH, SA, ZP, UK), data collection (MK, LH, ZP); statistical analysis (MK, LH).
